# Sex-based differences and relationship with the restricted knee flexion angle due to aging: a comparative study

**DOI:** 10.1186/s12891-023-06367-0

**Published:** 2023-05-04

**Authors:** Hiroshi Ohko, Susumu Ota

**Affiliations:** Department of Rehabilitation and Care, Seijoh University, 2-172 Fukinodai, Tokai, 476-8588 Aichi Japan

**Keywords:** Knee, Aging, Sex, Reliability, Range of motion.

## Abstract

**Background:**

The relationship between inferior patellar mobility (IPM) and knee flexion angle has yet to be elucidated. This study aimed to develop quantitative IPM measurement methods and clarify the relationship between IPM and knee flexion angle in community-dwelling older females.

**Methods:**

This was a cross-sectional study. Overall, 128 healthy older women (age 65–79 years) were recruited from the community to evaluate the relationship between IPM and knee flexion angle. This study was performed between May 2015 and December 2017. The reference value of and sex differences in IPM were evaluated in 205 healthy young adults aged between 19 and 21 years. IPM was compared between healthy older and young women and was objectively measured using our specially designed patellofemoral arthrometer (PFA). Patellar mobility was calculated by normalization to body height. IPM reliability was assessed before all measurements.

**Results:**

Intraclass correlation coefficients for intratester and intertester reliabilities varied from 0.87 to 0.99. The normal range based on two standard deviations of inferior patellar displacement/body height was 5.9–13.5% (young men), 5.1–14.3% (young women), and 1.2–8.8% (older women). IPM was significantly lower in older than young women (*P* < 0.001). There was a significant positive correlation (*r* = 0.72 and *P* < 0.01) between IPM and knee flexion angle in healthy older women unable to flex the knee joint fully.

**Conclusions:**

Our PFA has good intratester and intertester reliability. The results suggest that IPM decreases with aging in women. IPM and knee flexion angle are correlated among older women unable to flex the knee joint fully.

**Clinical trial registration:**

Not applicable.

**Supplementary Information:**

The online version contains supplementary material available at 10.1186/s12891-023-06367-0.

## Background

A decrease in the knee joint’s range of motion (ROM) directly affects activities of daily living, including walking, using stairs, rising from a chair, and wearing or removing undergarments; therefore, it is important to maintain and improve ROM [1–3]. A decrease in the ROM of knee flexion is associated with osteoarthritis [4], stiffness after total knee arthroplasty (TKA) [5, 6], postoperative arthrofibrosis [7], and other complications. Moreover, bed rest [8], shortening of the rectus femoris muscle [9], and soft tissue changes [10], including adhesion, can occur because of or in tandem with underlying conditions.

According to Panni et al. [11] and Dragoo et al. [12], reduced patella mobility is associated with limited knee flexion, especially in inferior patella mobility. Additionally, Jerosch and Aldawoudy [13] showed that the knee flexion angle could be improved in patients who undergo TKA by removing tissue adhesions around the patella, including the suprapatellar pouch and intra-articular fibrous bands. Although some reports claim that inferior patellar mobility (IPM) is related to limited knee joint flexion [14–16], such mobility has only been measured subjectively, owing to the absence of a quantitative measurement method. The patellar gliding test, conducted by manually moving the patella in the medial, lateral, and inferior direction to the end range of movement, is commonly used to measure passive patellar mobility [14]. The Cincinnati Knee Rating System and Kolowich (quadrant) method, which measure patellar mobility using the patellar sliding test, provide a subjective assessment of patellar movement [17–19]. Thus, mobility assessment of the patella has been subjectively evaluated in the past.

Ota et al. [20, 21] developed the patellofemoral arthrometer (PFA), which enabled reliable quantitative measurements of medial and lateral patellar mobility. They used the PFA to investigate the knee flexion angle and medial and lateral (but not inferior) patellar mobility of patients post-TKA. They found that these parameters were not related horizontally [22]. Thus far, the objective measurement of medial and lateral patellar mobility has been established by Ota et al.; however, the relationship between knee flexion angle and IPM, which are suspected to be related clinically, has not yet been clarified because no objective method to measure IPM has been established. Establishing such a quantitative evaluation method will help determine if a relationship exists between IPM and knee flexion angle. If so, this would validate that improving IPM may be a therapeutic approach to improving knee joint flexion. Thus, this study aimed to show that the knee flexion angle of older women with limited knee flexion was associated with decreased IPM. The healthy older people recruited in this study were women with a high prevalence of knee osteoarthritis who were expected to have reduced knee flexion angles.

The purposes of the present study were as follows: (1) to confirm the reliability of IPM using PFA in young, healthy participants, as well as (2) sex differences; and (3) to examine age-related changes in IPM in young, healthy participants and healthy older women, as well as (4) the relationship between IPM and knee joint flexion angle in healthy older women.

## Methods

### Study participants

This observational study was performed from May 2015 to December 2017. A total of 205 pain-free students from Seijoh University, Japan, volunteered for the study; among these students, 103 were men (age: 20.3 ± 1.5 years; height: 170.8 ± 5.5 cm; body mass index [BMI]: 22.0 ± 3.3 kg/m^2^), and 102 were women (age: 20.6 ± 2.2 years; height: 158.2 ± 5.1 cm; BMI: 20.6 ± 2.2 kg/m^2^). Healthy older adults were recruited from among those who had previously attended a lecture at Seijoh University, and older women aged 65 years or older (*n* = 128; age: 72.2 ± 6.7 years; height: 150.4 ± 4.6 cm; BMI: 22.8 ± 3.7 kg/m^2^) were recruited from the local community. The exclusion criteria were having a history of knee pathologies and/or testing positive on a clinical patellar test, such as Clarke’s or patellar femoral grinding test. We randomly chose the right or left patella for each participant by flipping a coin and subsequently assessed its mobility. All subjects had the knee flexion angle measured by an experienced physical therapist using a goniometer. All participants were informed of the nature of the study before they provided written informed consent. This study was conducted in accordance with the principles embodied in the Declaration of Helsinki and was approved by the Ethics Committees of Seijoh University, Japan.

## Assessment of patellar mobility

### Instrumentation

**Fig. 1:**
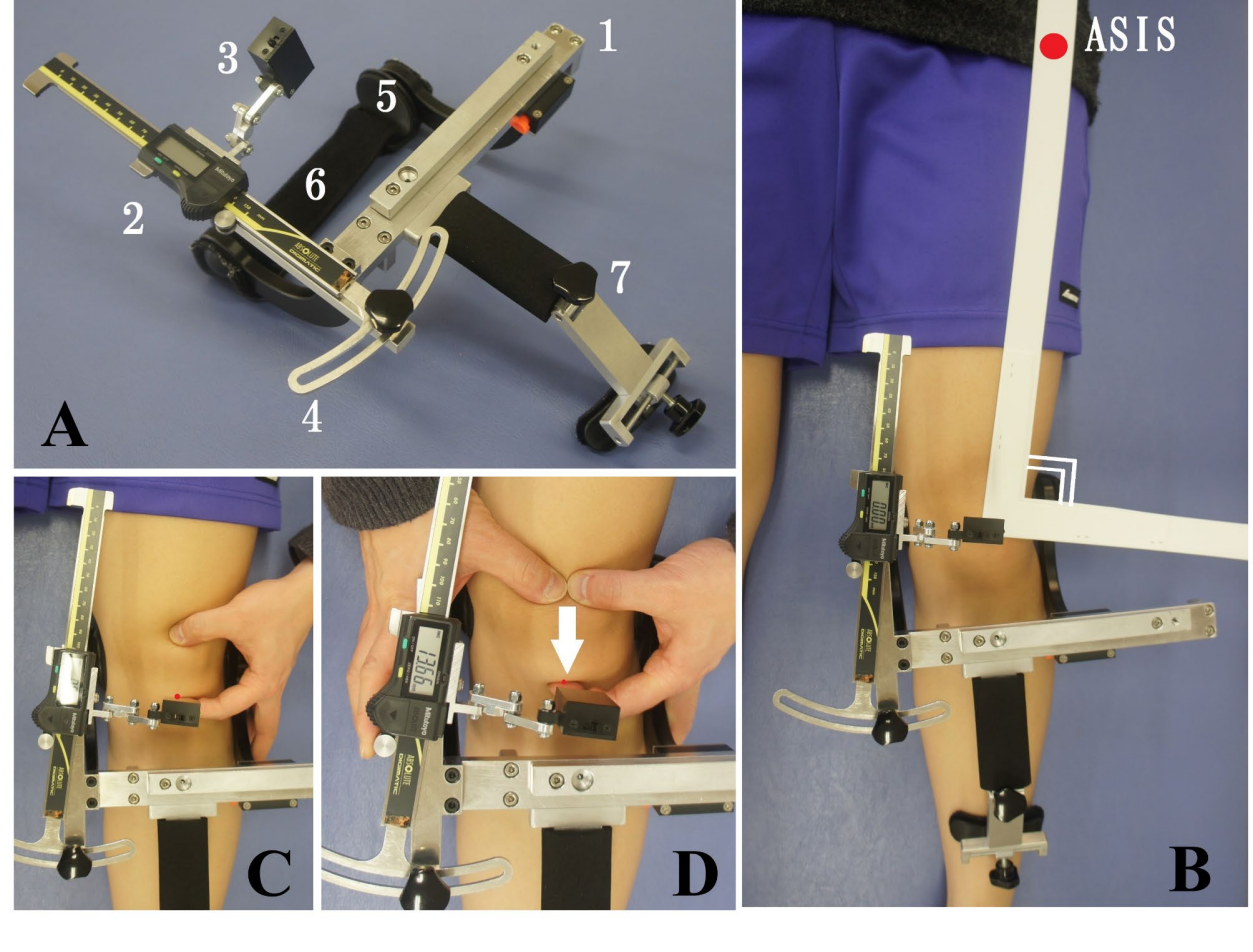
Components of the patellofemoral arthrometer and measurement method of inferior patellar mobility. (a) Components of the patellofemoral arthrometer. (1) Base, (2) digital caliper, (3) adjustable laser module arm, (4) plane adjuster, (5) clamping mechanism, (6) thigh strap, and (7) fixed arm. (b) The patellofemoral arthrometer can be clamped to the femoral condyles. The plane adjuster allows the digital caliper to lie parallel to the imaginary line between the center of the patella and the anterior superior iliac spine (ASIS). (c) The patellar apex was located using palpation and aligned to the laser of the adjustable laser module arm. This was considered the starting position for patellar mobility measurements, with the digital caliper set to 0 mm. (d) IPM was measured as the difference between the starting position (patellar apex) and the position following the application of an 80 N of force inferiorly

To assess IPM, a modified PFA with added superior and inferior patellar measurements (Brace Fit Co., Ltd., Aichi, Japan) was used with a digital caliper (Fig. 1a). The PFA was designed to measure patellar displacement in millimeters during the initial (fixed) frontal plane motion (medial, lateral, superior, and inferior translation).

### Procedures

Data from the previous study were used to determine the evaluation posture of study participants (i.e., 0° hip rotation, supine position) [21, 22]. The PFA was clamped to the femoral condyles and firmly secured parallel to the bed by fastening it to the thigh with a strap. The digital caliper was positioned parallel to an imaginary line connecting the medial patella to the anterior superior iliac spine (Fig. 1b). Before each test, the level of pushing force (approximately 80 N) was confirmed with a handheld MicroFET2 dynamometer (Hoggan Health Industries Inc., West Jordan, UT, USA). The assessment confirmed leg muscle relaxation by palpating the quadriceps and passively moving the patella in the medial and lateral directions.

A pilot study was performed in which the patellar displacement was measured in 20 participants (10 men and 10 women). There were significant differences in IPM on the third and subsequent measurements, and stable values were obtained (first vs. second: *P* = 0.01, second vs. third: *P* = 0.04, and third vs. fourth: *P* = 1.0). Thus, the measurement was performed after mobility training had been conducted at least three times.

To measure IPM, the patellar apex was palpated and located with a laser using the adjustable laser module arm. At that point, the digital caliper was set to zero (i.e., the initial position) (Fig. 1c). The inferior displacement of the patella was subsequently determined by manually pushing the patella inferiorly (at 80 N). At that point, the patellar apex was again located by sliding the laser module arm on the caliper and reading the measurement (Fig. 1d). IPM was performed three times. The average value was used for statistical analysis. Because displacement is thought to be affected by body size, the degree of displacement was normalized to body size as represented by height (HT) [23]. Patellar mobility was adjusted for HT (patellar mobility/HT × 100). The absolute values of inferior patellar displacement (IPD) and IPD/HT were used to compare patellar mobility between young men, young women, and older women.

## Reliability study

Twenty-nine young pain-free individuals (15 men and 14 women) participated in the reliability study of IPD. The average age, HT, and BMI of participants (± standard deviation [SD]) were 21.5 ± 0.6 years (21.7 ± 0.6 years in men, 21.4 ± 0.5 years in women), 166.5 ± 5.2 cm (173.5 ± 5.3 cm in men, 159.5 ± 5.2 cm in women), and 21.5 ± 0.6 kg/m^2^ (22.1 ± 1.7 kg/m^2^ in men, 19.9 ± 1.7 kg/m^2^ in women), respectively. Intra- and inter-tester reliability when quantifying IPM was determined based on measurements made by two testers on different days.

Before data collection, testers 1 and 2 practiced measurements on 10 participants for 100 min. Subsequently, the testers randomly measured patellar mobility in the same session in each study participant. Tester 1 performed the measurements in both testing sessions, which were at least three days apart for intratester reliability. Tester 2 assessed the patellar mobility on day 1 only to determine intertester reliability. The testers were blinded to all measurements.

The intra- and intertester reliability of IPD readings obtained with the PFA were assessed using intraclass correlation (ICC). The standard error of the mean (SEM) was calculated using the following equation: SEM = SD × (√1 – ICC). After the reliability study, the patellar mobility study commenced using the same procedures as in the practice session and reliability study. To further investigate the actual patellar displacement beyond measurement errors, the smallest real difference (SRD) was used to indicate the magnitude of change that would exceed the expected trial-to-trial variability. The SRD was calculated using the following equation: 1.96 ×√2 × SEM^2^. The ICC, SEM, and SRD values are presented in Table 1.

**Table 1:** Reliability of patellar mobility measurements using a patellofemoral arthrometer (*n* = 29)

	Intratester			Intertester		
	ICC (95% CI)	SEM	SRD	ICC (95% CI)	SEM	SRD
IPD (mm)	0.99 (0.98–0.99)	0.29	0.80	0.87 (0.73–0.94)	1.12	3.14

ICC, intraclass correlation coefficient; CI, confidence interval; IPD, inferior patellar displacement; SEM, standard error of the mean; and SRD, smallest real difference.

### Data analysis

The Shapiro–Wilk test was used to test for normality. Unpaired *t*-tests were used to compare patellar mobility between young men and women and between young and older women. For comparisons between the three groups, a one-way analysis of variance was performed with the Tukey Kramer post-hoc method. A *P*-value of < 0.05 was considered statistically significant.

Healthy older women were classified into two groups based on whether they were able to sit in seiza (a Japanese style of kneeling with the buttocks resting on the turned-out heels); 92 women were able to flex the knee joint fully (the seiza-possible group), and 36 were unable to do so (< 150°) (the seiza-impossible group). There were no significant differences in physical characteristics between the two groups of healthy older women. The correlation between patellar mobility and knee flexion angle was assessed in each group using Pearson’s correlation coefficient. All statistical analyses were performed using SPSS version 25.0 (IBM Corp., Tokyo, Japan).

## Results

The intratester reliability of IPD measurements was 0.99 (95% confidence interval [CI]: 0.98–0.99), with an SEM of 0.29 mm and SRD of 0.80 mm. The intertester reliability of IPD measurements was 0.87 (95% CI: 0.73–0.94). The SEM and SRD were 1.12 and 3.14 mm, respectively (Table 1). Table 2 presents the absolute values of IPD measurements in young men (IPD: 16.5 ± 3.3 mm; IPD/HT: 9.7 ± 1.9%) and women (IPD: 15.4 ± 3.7 mm; IPD/HT: 9.7 ± 2.3%), as well as in older women (IPD: 7.5 ± 2.8 mm; IPD/HT: 5.0 ± 1.9%). IPD/HT values normalized for body height are also shown. A significant difference in IPD was found between young men and women (*P* = 0.02; 95% CI: 0.17–2.11). However, the normalized IPD (IPD/HT) did not significantly differ between sexes (*P* = 0.85; 95% CI: -0.64–0.53). Thus, only absolute displacement showed sex-based differences in IPD.

**Table 2:** Descriptive data for patellar mobility in young, healthy men and women

	Total (*n = 205*)	Men (*n = 103*)	Women (*n = 102*)	*P-*value (95% CI)
IPD (mm)	15.9 ± 3.6	16.5 ± 3.3	15.4 ± 3.7	*P* = 0.02 (0.17, 2.11)
IPD/HT (%)	9.7 ± 2.1	9.7 ± 1.9	9.7 ± 2.3	*P* = 0.85 (-0.64, 0.53)

CI, confidence interval; IPD, inferior patellar displacement; and HT, height.

Values are presented as mean ± standard deviation.

We defined the normal patellar mobility range as 2 SD within a specific group. In our sample, the normal IPDs in young men and women were 9.9–23.1 mm and 8.0–22.8 mm, respectively. Using the same definition, the normal IPD/HT ranges were 5.9–13.5% in young men and 5.1–14.3% in young women. Similarly, the IPD range among older women was 1.9–13.1 mm, whereas the IPD/HT range was 1.2–8.8%.

Absolute and corrected values for IPD were significantly lower in the seiza-possible group (7.8 ± 2.6 mm, 5.2%±1.7%) and seiza-impossible group (6.7 ± 3.3 mm, 4.5%±2.2%) than in young women (15.4 ± 3.7 mm, 9.7%±2.3%) (*P* < 0.001) (Table 3).

**Table 3:** Descriptive data for patellar mobility in young women and older women

		Older women		*P*-value (95% CI)	
	Young women (*n = 102*)	*Seiza*-possible (*n = 92*)	*Seiza*-impossible (*n = 36*)	Young women vs. *seiza-*possible	*Seiza-*possible vs. *seiza-*impossible
IPD (mm)	15.4 ± 3.7	7.8 ± 2.6	6.7 ± 3.3	*P <* 0.001 (0.16, 1.25)	*P =* 0.044 (0.03, 2.20)
IPD/HT (%)	9.7 ± 2.3	5.2 ± 1.7	4.5 ± 2.2	*P <* 0.001 (0.18, 1.36)	*P =* 0.02 (0.02, 1.47)

The “seiza” possible and impossible groups comprised participants who were able to flex the knee joint fully and those who were unable to do so (< 150°), respectively.

CI, confidence interval; IPD, inferior patellar displacement; and HT, height.

Values are presented as mean ± standard deviation.

**Fig. 2:**
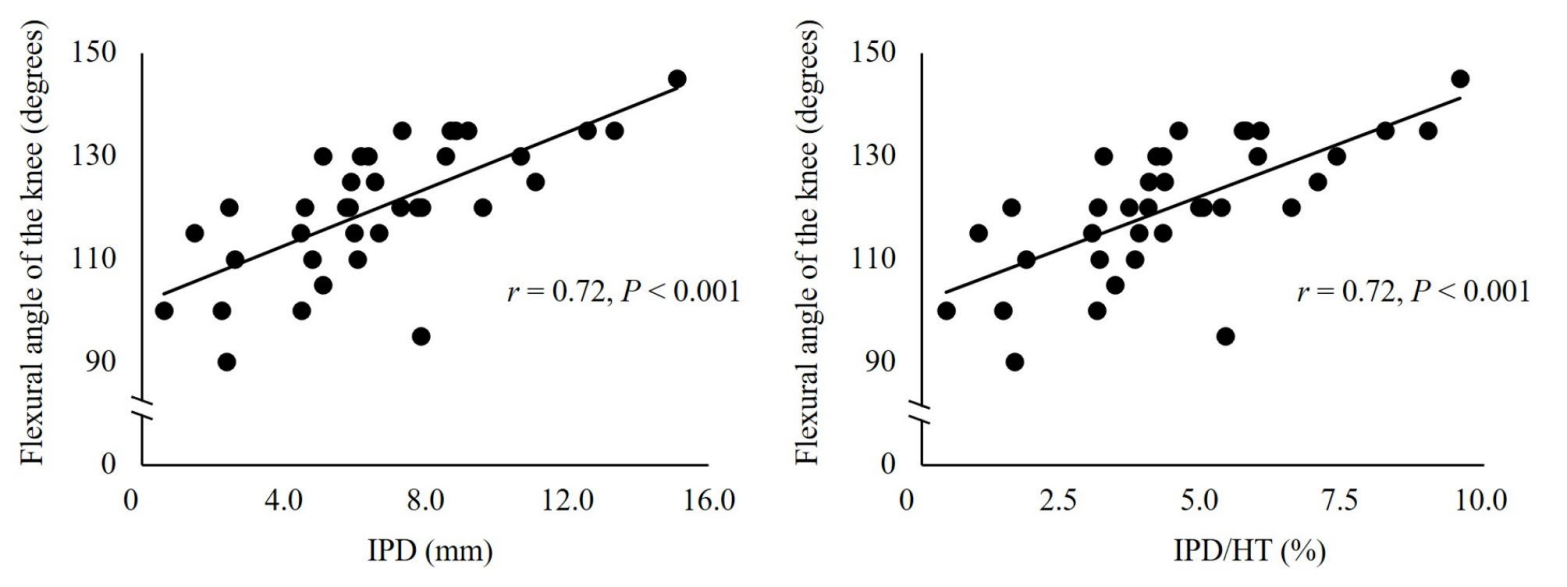
Relationship between inferior patellar mobility and knee flexion angle among older women. The correlations between patellar mobility and knee flexion angle in the seiza-possible and seiza-impossible groups are assessed using Pearson’s correlation coefficient IPD, inferior patellar displacement; HT, height

Furthermore, IPM in the seiza-impossible was significantly lower than that in both young women and the seiza-possible group in both adjusted and unadjusted models (*P* = 0.044). Additionally, in the models mentioned above, there was a significant positive correlation (*r* = 0.72, *P* < 0.001) between IPM and the knee flexion angle in the seiza-impossible group (Fig. 2).

## Discussion

The main findings of this study are as follows: there was a significant difference in the absolute value of IPD between young men and women, but when adjusted for body height, the difference became insignificant. Absolute and normalized values for IPD were significantly lower in older women than in young women. Moreover, the seiza-impossible group of older people had significantly lower IPD than the seiza-possible group and young women. Additionally, in the seiza-impossible group of older people, there were significant positive correlations between the absolute and normalized values of IPD and knee flexion angle. The newly developed modified PFA with inferior patellar measurement can measure IPM with good intra- and inter-examiner reliability (ICC, 0.87–0.99) and is suitable for clinical application.

Methods for evaluating the patella include tracking [24], magnetic resonance imaging (MRI), computed tomography, conventional radiography in the static position [25–28], and medial and lateral mobility assessment [20–23]. However, they all measure the position of the patella in a static state. Until now, dynamic mobility assessment of the patella has been measured by subjective methods such as the Cincinnati Knee Rating System and Kolowich (quadrant) method [14, 17]. Clinically, a relationship between IPM and knee joint flexion angle is suspected [11, 12, 14, 29], but this has been unclear because the measurement of IPM by an objective method has not been established. Modified PFA can be measured and evaluated as an objective value in a manner consistent with actual patellar mobility assessment in the clinic, and it may clarify the relationship between knee flexion angle and IPM. In addition, it is easy to wear and measure, and we believe that it will become a new measurement method with sufficient clinical application. Additionally, using the mean ± 2 SDs (i.e., 94.2% of normal distribution among men and 94.6% of that among women), it was necessary to establish the mean IPM in young men and women and adjust for normalized body height as described in previous reports [20]. In this study, we did not measure IPM in older men, so we consider that a comparison of patella mobility between older men and women is also necessary in the future.

The IPM values among both the seiza-possible and seiza-impossible groups of older women were significantly lower than those among young women, with both older people groups showing half the mobility of their young counterparts. Diminished muscle flexibility and increased soft tissue stiffness due to aging have been demonstrated in humans [30]. Notably, decreased soft tissue and muscle flexibility near the base of the patella and distal thigh may affect inferior patella mobility [11–13]. IPM was significantly lower in the seiza-impossible group than in the seiza-possible group. Furthermore, there was a significant correlation between IPM and knee flexion angle in the seiza-impossible group, suggesting that IPM influenced knee flexion angle. Seiza is a traditional Japanese way of sitting, but in recent years, changes in lifestyle do not require people to sit on their knees anymore. However, a decrease in knee flexion angle is a risk factor for knee OA [31], which may be related to deep knee bending movements such as Seiza. Further research is needed to clarify this.

Suprapatellar pouch lesion is considered a cause of IPM. The patella and suprapatellar pouch are connected, and the adhesion of these two components may cause pain, flexion contractures, and stiffness [13, 32], which may reduce patellar mobility. Kapandji [33] also reported that when knee flexion increases from 90° to more than 135°, the suprapatellar pouch becomes completely “unpleated” (i.e., straight without creases). The function of the suprapatellar pouch during knee flexion has been reported to be anatomically important; hence, IPM may be caused by diminished flexibility of soft tissues around the knee, including the suprapatellar pouch [11].

The relationship between the flexibility of soft tissues around the knee and IPM reportedly plays a role in some diseases that cause limited knee joint flexion. In the case of knee osteoarthritis, it has recently been suggested that joint inflammation is confined to the articular cartilage of the knee and affects the surrounding soft tissues, leading to peripatellar lesions and knee stiffness [34]. In patients with knee joint fibrosis, the signs and symptoms of arthrofibrosis include reduced patellar mobility, diminished knee ROM, tenderness around the knee, pain, and atrophy of the quadriceps. IPM affects diminished knee flexion ROM [35–39]. Furthermore, in patients with knee joint fibrosis, lesions around the patella, including the infrapatellar fat pad, pretibial recess, anterior interval, and suprapatellar pouch, may arise [40], affecting IPM.

The causative factors of IPM have yet to be determined because of the lack of methods that can objectively evaluate it. Moving forward, it is necessary to clarify the relationship between IPM and soft tissue flexibility around the knee in patients with limited knee flexion. If the limits of IPM are identified, improvements in such mobility using therapeutic interventions can lead to an increased knee flexion angle.

This study has some limitations. First, the study comprised healthy participants, and patellar mobility was not compared in the patient population. In future studies, it would be necessary to examine patellar mobility in patients with knee osteoarthritis. Second, we did not compare our results with those obtained using gold-standard procedures (i.e., MRI or X-ray fluoroscopy) for validation. Ota et al. [22] evaluated the mobility of the medial and lateral patella with MRI using conventional PFA and obtained good results. Moreover, the position of the patella at rest needs to be specifically examined as it may be related to the inferior mobility of the patella. Specifically, it is necessary to evaluate the Insall-Salvati ratio using radiography [26]. Third, we did not quantify the force that pushes down the patella; rather, we used a dynamometer that we practiced pushing at 80 N before measurement. However, it was unclear whether the optimal force was applied to push the patella downward in actual measurements. To solve this problem, it is necessary to consider obtaining measurements while pressing back the patella in real-time using a sheet sensor, for example.

## Conclusions

The PFA we developed can measure IPM with good intra- and inter-examiner reliability. Our results suggested that IPM decreases with age in women. The mobility of the inferior patella in unable seiza was significantly lower in older women unable to sit in seiza. Moreover, the knee flexion angle and IPM correlated with older women unable to sit in seiza.

## Electronic supplementary material

Below is the link to the electronic supplementary material.

Supplementary Material 1

## Data Availability

The datasets generated and/or analyzed during the current study are not publicly available due to patient privacy concerns but are available from the corresponding author on reasonable request.

## Abbreviations

CI: Confidence interval
HT: Height
ICC: Intraclass correlation coefficient
IPM: Inferior patellar mobility
PFA: Patellofemoral arthrometer
ROM: Range of motion
SEM: Standard error of the mean
SRD: Smallest real difference
TKA: Total knee arthroplasty
MRI: Magnetic resonance imaging
